# Investigating the Temporal Patterns within and between Intrinsic Connectivity Networks under Eyes-Open and Eyes-Closed Resting States: A Dynamical Functional Connectivity Study Based on Phase Synchronization

**DOI:** 10.1371/journal.pone.0140300

**Published:** 2015-10-15

**Authors:** Xun-Heng Wang, Lihua Li, Tao Xu, Zhongxiang Ding

**Affiliations:** 1 College of Life Information Science and Instrument Engineering, Hangzhou Dianzi University, Hangzhou, 310018, China; 2 Department of Radiology, Zhejiang Provincial People's Hospital, Hangzhou,310014, China; Wake Forest School of Medicine, UNITED STATES

## Abstract

The brain active patterns were organized differently under resting states of eyes open (EO) and eyes closed (EC). The altered voxel-wise and regional-wise resting state active patterns under EO/EC were found by static analysis. More importantly, dynamical spontaneous functional connectivity has been observed in the resting brain. To the best of our knowledge, the dynamical mechanisms of intrinsic connectivity networks (ICNs) under EO/EC remain largely unexplored. The goals of this paper were twofold: 1) investigating the dynamical intra-ICN and inter-ICN temporal patterns during resting state; 2) analyzing the altered dynamical temporal patterns of ICNs under EO/EC. To this end, a cohort of healthy subjects with scan conditions of EO/EC were recruited from 1000 Functional Connectomes Project. Through Hilbert transform, time-varying phase synchronization (PS) was applied to evaluate the inter-ICN synchrony. Meanwhile, time-varying amplitude was analyzed as dynamical intra-ICN temporal patterns. The results found six micro-states of inter-ICN synchrony. The medial visual network (MVN) showed decreased intra-ICN amplitude during EC relative to EO. The sensory-motor network (SMN) and auditory network (AN) exhibited enhanced intra-ICN amplitude during EC relative to EO. Altered inter-ICN PS was found between certain ICNs. Particularly, the SMN and AN exhibited enhanced PS to other ICNs during EC relative to EO. In addition, the intra-ICN amplitude might influence the inter-ICN synchrony. Moreover, default mode network (DMN) might play an important role in information processing during EO/EC. Together, the dynamical temporal patterns within and between ICNs were altered during different scan conditions of EO/EC. Overall, the dynamical intra-ICN and inter-ICN temporal patterns could benefit resting state fMRI-related research, and could be potential biomarkers for human functional connectome.

## Introduction

Previous studies found that the scan conditions of eyes-open (EO) or eyes-closed (EC) might have impacts on resting state functional connectivity [1]. Different active patterns were found in visual cortex under scan conditions of EO/EC [2]. Through voxel-level measures, the different dynamical attributes of spontaneous brain activities during EO/EC were discovered by the amplitude of low frequency fluctuations (ALFF) [1, 3–5]. Through regional-level measures, the characteristic resting states regional-wise fractal ALFF during EO/EC could be decoded via support vector machines (SVMs) [6]. Moreover, distinctive patterns of EO/EC were found with measures of regional ALFF and functional connectivity, and the two measures were highly correlated [7]. Thus resting states of EO/EC might influence dynamic functional connectivity. Neurophysiological and identical alpha-band activities of MEG signals were related to resting states of EO/EC [8]. In addition, reorganized functional networks were found between EO/EC across different frequency bands of MEG signals, suggesting the complexity of brain dynamics [9]. Although the directional properties of interactions among large-scale brain networks between EO/EC have been investigated by Gaussian Bayesian network [10], the dynamical patterns of brain networks under EO/EC still remain largely unexplored.

The couplings of neural signals might fluctuate over time in dynamical ways [11], suggesting the non-stationary nature of brain networks [12]. According to previous studies, the dynamical, or time-varying functional connectivity was usually measured via sliding-window techniques [11, 13]. Through time-varying method, the temporal variability of functional connectivity was discovered between certain brain regions, resulting in seven dynamical clusters [13]. Five micro-states (intrinsic functional connectivity patterns) were found for the temporal dynamics within posteromedial cortex [14]. Notably, the inter-regional dynamical functional connectivity might be modulated by resting state networks [15]. Therefore, resting state networks might play important roles in the dynamical mechanisms of human brain.

Resting state networks, also named as intrinsic connectivity networks (ICNs), are spatially independent large-scale brain networks, the oscillations within which are highly synchronized together [16, 17]. The time course of the ICN might reflect the mean spontaneous fluctuation within the corresponding network [18]. Moreover, the time courses of ICNs were related to physiological hemodynamic fluctuation [19]. The Hurst exponent for the time courses of DMN was related to personality trait [20]. The complexities for the time courses of ICNs exhibited identical patterns and were different from noise [21]. Therefore, the temporal patterns of ICNs might reflect certain biological meanings [18, 22]. Specially, the dynamic fluctuations within DMN might play key role in resting state mind-wandering [23]. The spontaneous activities within DMN might be modulated by different scan conditions of EO/EC [3]. Of note, resting states of EO/EC exhibited distinct brain activities in DMN-related brain regions [6]. Thus the temporal patterns of DMN might be related to the temporal patterns of other ICNs under EO/EC differently. Based on the time-courses of ICNs, the temporal patterns of ICNs contained two categories of features: 1) univariate features and 2) bivariate features. The univariate patterns might indicate the information flow within ICNs [24, 25]. The bivariate patterns might represent the information interactions between ICNs [26]. However, the relationships of dynamical temporal patterns within and between ICNs remain poorly understood.

Phase synchronization (PS), usually based on Hilbert transform, is a bivariate feature for coupled neural signals [27]. There were emerging studies on the synchronization of neural signals [28–30]. Mean phase coherence was applied as a measure of synchrony for EEG-related research [31]. Correlation between probabilities of recurrence was proposed to construct graphs for resting state fMRI datasets [32]. Moreover, concurrent EEG and resting state fMRI-related studies found the activity in fronto-parietal network was related to alpha-band phase synchrony [33]. Recently, instantaneous PS was used to investigate time-varying functional connectivity based on resting state fMRI [34]. However, little is known about the dynamical synchronization between ICNs. Mathematically, the PS was based on instantaneous phase difference of coupled time-series. In addition, the instantaneous amplitudes of time-series could be recognized as univariate features. Thus, instantaneous features based on Hilbert transform could provide a unified framework to investigate the univariate and bivariate temporal features of ICNs.

Based on the above studies, we reasoned that the ICNs might exhibit characteristic dynamical functional connectivity under resting states of EO/EC, and hypothesized that the altered dynamical temporal patterns could be discovered with resting state synchrony. To this end, a cohort of health subjects with resting state fMRI datasets of EO/EC states were recruited from the 1000 Functional Connectomes Project. First, the time-courses of ICNs were extracted from the spatially normalized 4D functional volumes. Then, the micro-states of synchrony between ICNs were analyzed through unsupervised learning. Finally, the altered dynamical temporal patterns within and between ICNs were determined to explore the network mechanisms of EO/EC.

## Methods

### Participants and MRI protocols

A cohort of healthy controls (students) were recruited from Beijing Normal University in China [1]. All research involving human participants have been approved by the 1000 Functional Connectomes Project (http://fcon_1000.projects.nitrc.org/) and the ethics committee of Institutional Review Board of Beijing Normal University Imaging Centre for Brain Research. Each participant has provided written informed consent, which was approved by the ethics committee of Institutional Review Board of Beijing Normal University Imaging Center for Brain Research [1]. All data were scanned from a Siemens Trio 3.0 Tesla scanner, and could be publicly obtained from the 1000 Functional Connectomes Project [35]. For this study, each participant has one structural MRI scan and two resting state fMRI scans. One resting session was scanned with eyes closed, another resting session was scanned with eyes open. The resting state fMRI datasets were consisted of 240 standard EPI volumes for each session (TR = 2000 ms, TE = 30 ms, 3.1 mm × 3.1 mm × 3.5 mm, 8 mins). The structural MRI datasets were based on MPRAGE sequence (TR = 2530 ms, TE = 3.39 ms, 1.3 mm × 1mm × 1.3 mm) [1]. Two subjects were discarded for missing scan resting sessions, leaving 46 subjects (22 males and 24 females; mean age ± SD, 22.54 ± 2.18 years) for subsequent analysis.

### Data Preprocessing

All raw datasets were preprocessed and normalized into standard brain space through commands from FSL (www.fmrib.ox.ac.uk/fsl) and AFNI (afni.nimh.nih.gov). The structural datasets were skull-stripped, segmented and nonlinearly registered to standard brain. The resting state datasets were preprocessed by following steps: 1) discarded the first five volumes; 2) motion correction; 3) spatial smoothed using FWHM = 6 mm; 4) regressed out Friston-24 motion parameters [36–38], global signal, whiter matter signal, cerebral spinal fluid (CSF) signal, as well as linear and quantic trends; 5) temporal filtered (0.01–0.08 Hz) [39]; 6) resampled to 3 mm × 3mm × 3mm.

### Time-courses for ICNs

In this paper, we analyzed the temporal patterns of ten well-established ICNs, the spatial maps of which could be obtained from the website of BrainMap [18]. Based on the ICA decompositions of both resting state fMRI and task fMRI, the spatial components of ten well-established ICNs exhibited very close correspondence of architecture during resting and active states [18]. For this study, the templates of ICNs were extracted using over 7,000 activation-peak images from BrainMap, resulting in correlations with behavioral domain [18]. Moreover, the ten ICNs have been applied in previous studies [14, 21], suggesting the value of their usability in resting state research. The names of ICNs were listed in Table 1.

**Table 1:** Names of 10 ICNs.

index	Names of ICNs
ICN1	Medial visual network (MVN)
ICN2	Occipital visual network (OVN)
ICN3	Lateral visual network (LVN)
ICN4	Default mode network (DMN)
ICN5	Cerebellum (CBN)
ICN6	Sensorimotor network (SMN)
ICN7	Auditory network (AN)
ICN8	Executive control network (ECN)
ICN9	Right frontoparietal network (RFPN)
ICN10	Left frontoparietal network (LFPN)

**Fig 1:**
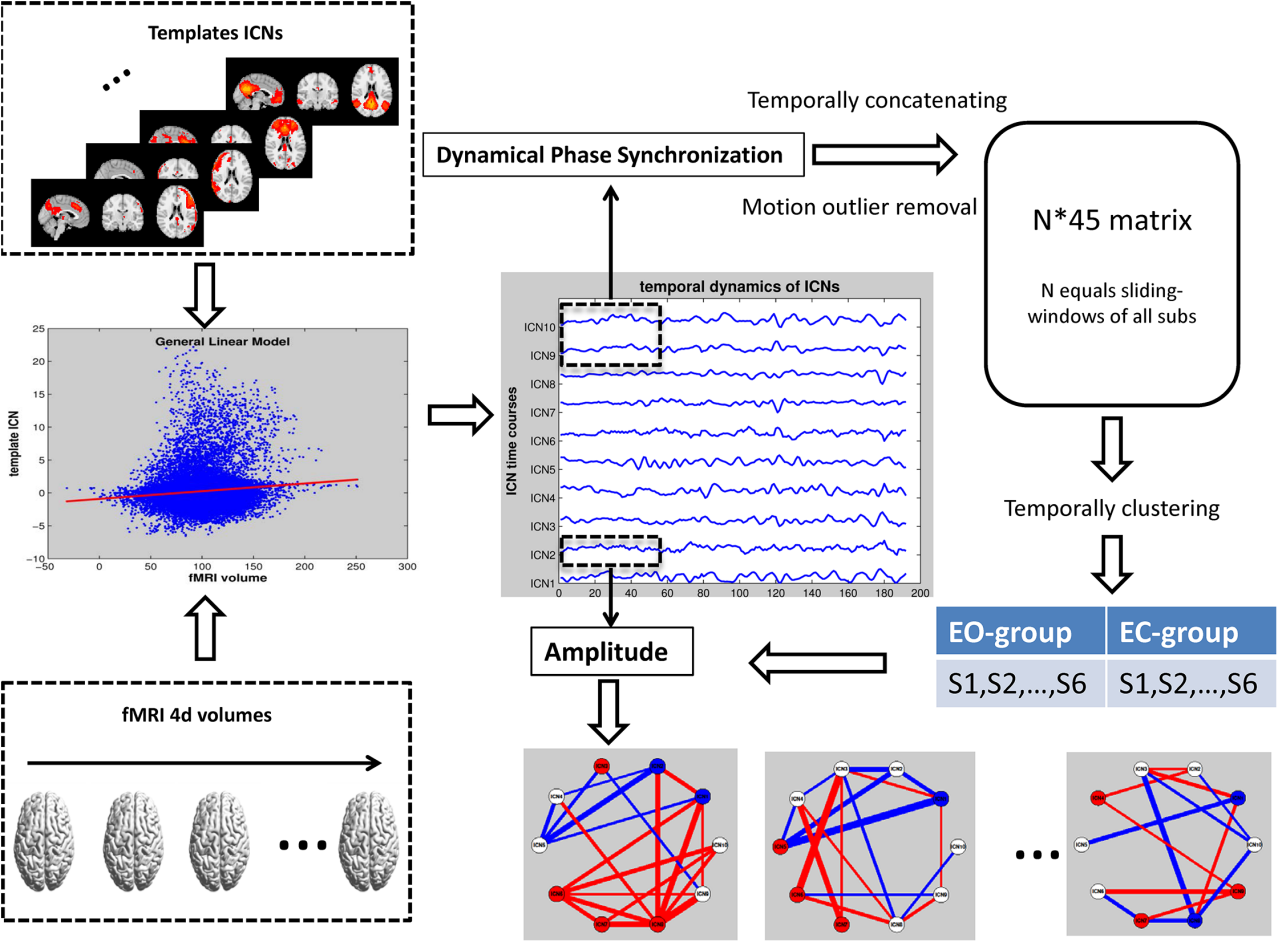
Data processing flowchart. The time-courses of ICNs are generated with GLMs. The time-varying amplitude and phase synchronization are obtained from Hilbert transform. After removing motion artifacts, the micro-states of dynamical synchrony are detected by hierarchical clustering analysis.

The time-course of an ICN could reflect the temporal dynamics within the network. To obtain the time-courses of ICNs, spatial general linear models (GLMs) were applied between the template ICNs and resting state fMRI volumes for each subject. The spatial GLMs procedures were carried out based on the first step of dual regression [24, 40], resulting an individual time-course of beta values for each ICN of each subject. The detailed information of spatial GLMs could be found in [24, 25, 40]. The flowchart of data processing could be found in Fig 1.

### Dynamical phase synchronization

Phase synchronization (PS) between coupled time-series could be obtained from the Hilbert transform of the analytic signals [27]. Hilbert transform was adopted in this paper for two reasons: 1) For a given neural signal, Hilbert transform could simultaneously produce instantaneous phases and amplitudes, which could be beneficial to investigating the univariate and bivariate features of time courses; 2) Hilbert transform could deal with non-stationary signals [41]. Given a time-course *s*(*t*), let $s\hat{(}t)$ equals the convolution of *s*(*t*) and 1 / (*πt*). The analytic signal can be denoted as $s^{\left(h\right)}(t)=s(t)+i\;s\hat{(}t)=A^{\left(h\right)}(t)e^{i\phi^{\left(h\right)}(t)}$. The instantaneous phase of *s*(*t*) can be defined as:

$$\phi^{\left(h\right)}(t)=\text{arctan}\frac{s\hat{(}t)}{s(t)}.$$

Given a sliding-widow on the instantaneous phases, the dynamical PS (mean phase coherence) [27, 31] can be defined as:

$$dPS=sqrt({\left[\frac{1}{m}\sum_{t=i}^{i+m}\text{cos}\;\hat{\varphi }(t)\right]}^{2}+{\left[\frac{1}{m}\sum_{t=i}^{i+m}\text{sin}\;\hat{\varphi }(t)\right]}^{2}),$$

where, $\hat{\varphi }(t)=\varphi_{1}(t)-\varphi_{2}(t)$, *i* =1,2,3,…,*n*, *i* is the index of sliding-window, *m* is the length of the sliding-widow, *n* is the number of sliding-windows. Here, the *φ*_1_(*t*) and *φ*_2_(*t*) represent the instantaneous phases of the coupled time courses of two ICNs, respectively.

In addition, the amplitude of the analytic signals was analyzed as univariate temporal patterns for ICNs. Given instantaneous amplitudes as $A^{\left(h\right)}(t)=abs(s(t)+i\;s\hat{(}t))$, the dynamical amplitude could be derived from the instantaneous amplitudes of the analytic signals in the following equation:

$$dAMP=\frac{1}{m}\sum_{t=i}^{i+m}A^{\left(h\right)}(t),$$

where *i* =1,2,3,…,*n*, *i* is the index of sliding-window, *m* is the length of the sliding-widow, *n* is the number of sliding-windows.

In order to comply with previous studies [13, 14], the length of the sliding-widow equals to 22 TRs (44s) for this study. The overlay of sliding-windows equals to 1 TR, thus each session could produce 213 sliding-windows before artifacts removal. The effects of sliding-widow length on the robustness of the results were analyzed additionally.

Moreover, two metrics were applied to investigate node properties of the graph consisted with ICNs. One metric is node amplitude or ICN amplitude, equals to dAMP within each sliding-window. Another metric is node strength or ICN strength, and could be defined as the average value of PS for the corresponding ICN to other ICNs within each sliding-window. Specially, the node properties for DMN were investigated by DMN amplitude and DMN strength.

### Motion outliers detection and removal

Given current concerns on motion artifacts in resting state fMRI datasets, frame-wise displacement (FD) and root-mean-square variance of the temporal derivative (DVARS) [36, 42] were applied to detect motion outliers in the functional volumes after motion correction. Here, the volume-wise DVARS was computed by the FSL plugin. The normalized DVARS should be approximately 1 if there were no artifacts. The outliers of DVARS were detected by boxplots (outside 1.5 times the interquartile range above the upper quartile and bellow the lower quartile). A marked artifactual time-point was defined based on FD > 0.5 mm or DVARS out of boxplot of the entire time-course. The backward and forward time points of the artifactual time point were also marked as outliers. If a motion outlier presented in a sliding-window, then sampling points within the sliding-window were marked as the motion-corrupted fragments. After removing motion-corrupted fragments for each subject, the bivariate features of dynamical PS were temporally concatenated together for unsupervised learning.

### Hierarchical clustering analysis

In this paper, hierarchical clustering analysis (HCA) was applied to evaluate the dynamical synchrony between ICNs [14]. HCA is a connectivity based clustering method, and seeks to build a hierarchy of clusters. The selection of HCA was based on three reasons [43]: 1) HCA does not require a predefined number of clusters; 2) HCA can produce a dendogram for visualization of the relationships between clusters; 3) The cluster label of each instance was a fixed value for a given number of clusters. The procedure of HCA contained three steps: First, the distance between pairs of instances was defined by Euclidean distance; Second, a new cluster was obtained from two nearest sets of distance using Ward's criterion as the linkage criterion [44]; Finally, all instances were agglomerated into one single cluster by repeating the above steps.

The number of clusters for micro-states was detected via the mclust package [45]. The procedure of detecting the optimized number of clusters contained the following steps: 1) temporally concatenated dynamical features of all subjects into a feature matrix; 2) random selected 200 instances from the feature matrix; 3) detected the number of clusters using model-based clustering embedded in mclust package [45]; 4) selected the optimized number of clusters based on the histogram of 1000 simulations of step 3.

After clustering analysis, the labeled instances were divided into two groups: EO-group and EC-group. For each cluster/state, formal testing was applied to investigate the altered temporal patterns of ICNs under EO/EC: 1) using t-test to find the altered univariate as well as bivariate features of ICNs; 2) using Fisher-Z transform to find the altered relationships between univariate and bivariate features of ICNs. More specifically, the test of difference between two correlations of independent groups contained the following steps: 1) obtaining the correlation coefficients for each group; 2) applying Fisher r-to-z transform on the correlation coefficients of the two groups, *Z*_1_ = (ln(1+*r*_1_)−ln(1−*r*_1_)) / 2, and *Z*_2_ = (ln(1+*r*_2_)−ln(1−*r*_2_)) / 2, where r_1_ and r_2_ represented the correlation coefficients of the two groups respectively; 3) deriving the Z value for difference between two groups,

$$Z=\frac{Z_{1}-Z_{2}}{\sqrt{\frac{1}{n_{1}-3}+\frac{1}{n_{2}-3}}}\;,$$

where Z_1_ and Z_2_ represented the Z-transformed correlations coefficients for the two groups respectively, n_1_ and n_2_ represented the numbers of instances in the two groups respectively [46].

## Results

### Clusters of dynamical phase synchronization between ICNs

**Fig 2:**
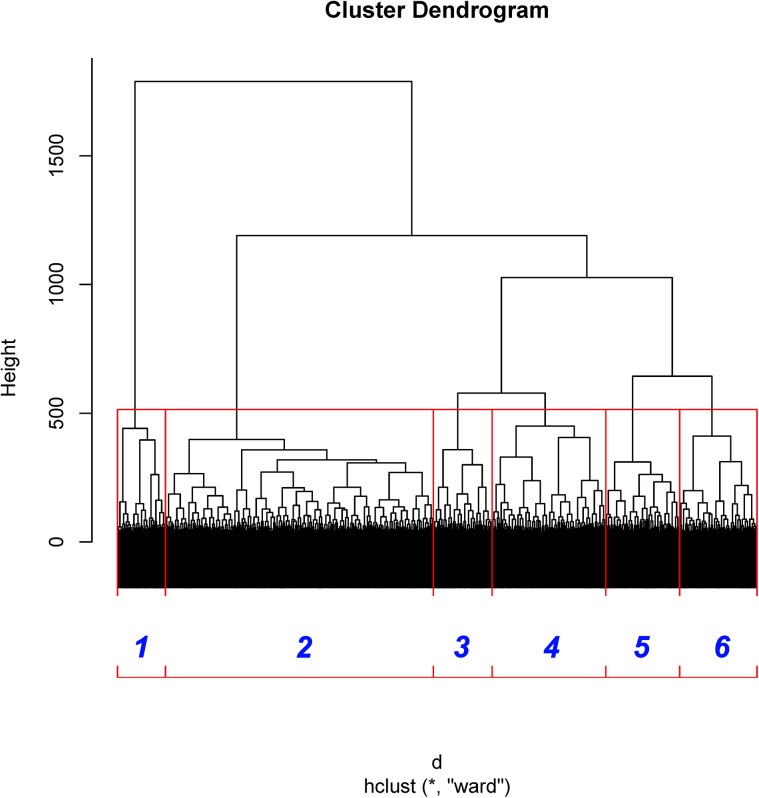
Dendogram of clustering of dynamical inter-ICN PS. The red rectangles denote the boundaries of clusters. The blue numbers denote the indices of clusters.

**Fig 3:**
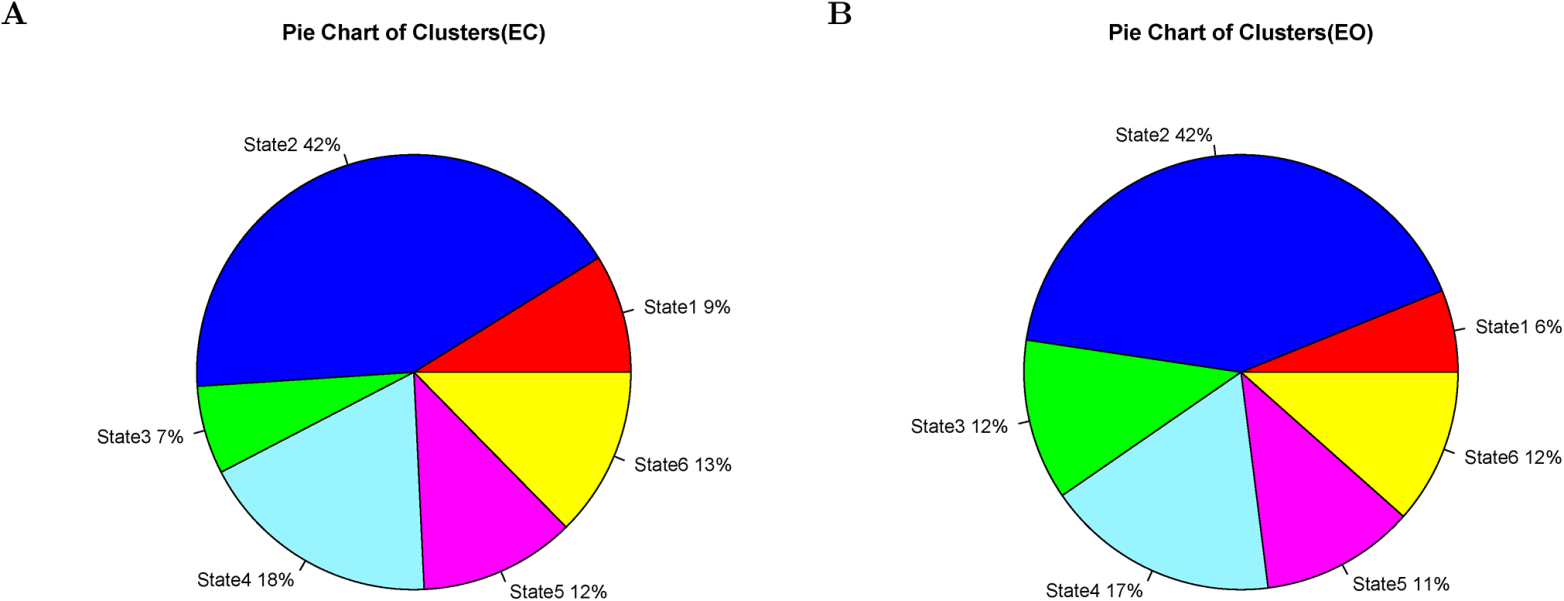
Percentages of clusters of dynamical inter-ICN PS. Subfigure A denotes the pie chart of clusters for eyes-closed resting state. Subfigure B denotes the pie chart of clusters for eyes-open resting state. The red, blue, green, baby blue, pink, and yellow colors denote clusters 1, 2, 3, 4, 5, and 6, respectively.

**Fig 4:**
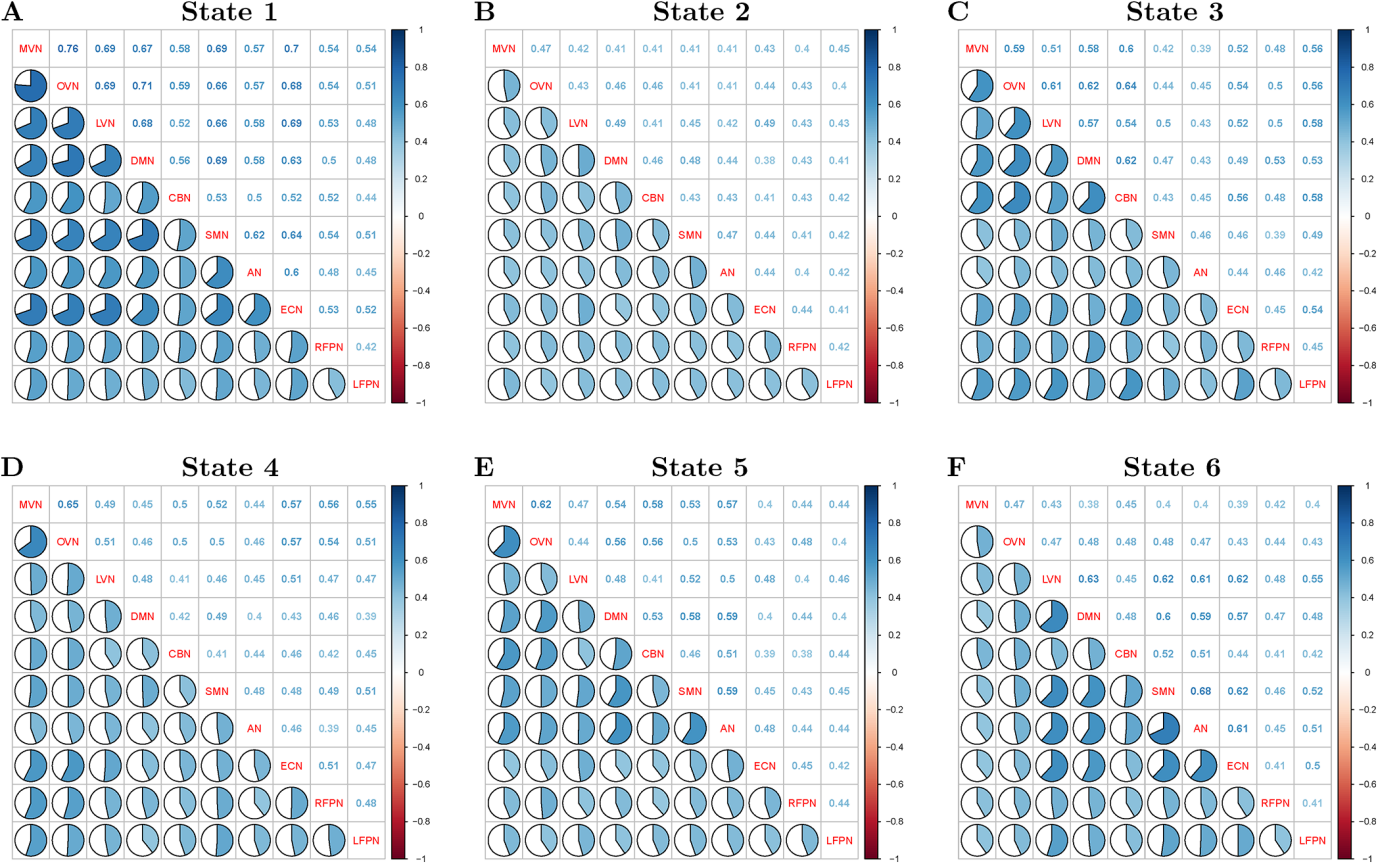
Mean dynamical inter-ICN PS of each cluster. In each subfigure, the names of ICNs are listed in the diagonal line. Subfigures A, B, C, D, E and F denote states 1, 2, 3, 4, 5, and 6, respectively.

**Fig 5:**
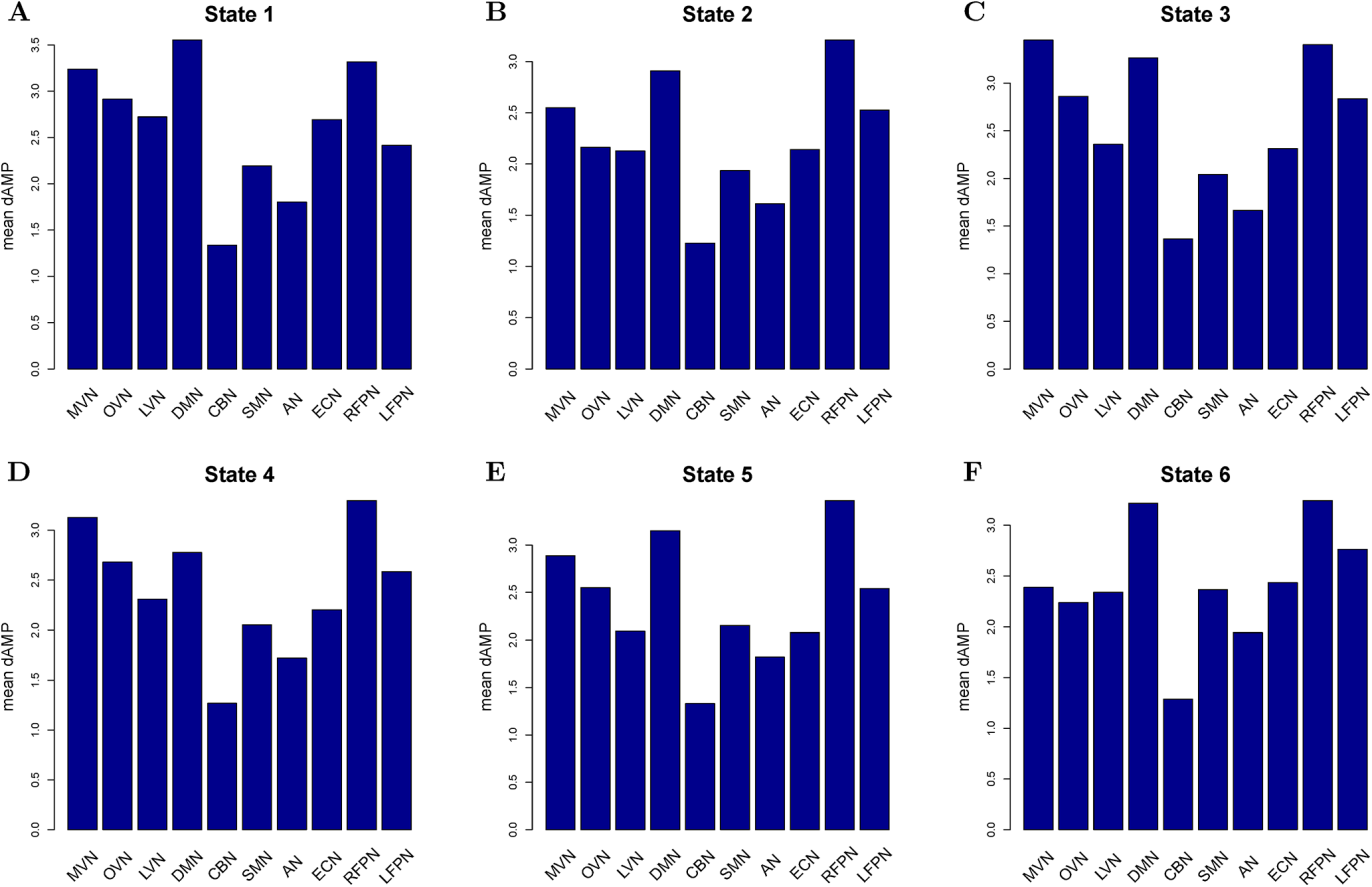
Mean dynamical intra-ICN amplitude of each cluster. Subfigures A, B, C, D, E and F denote states 1, 2, 3, 4, 5, and 6, respectively.

Fig 2 shows the dendogram of clustering. Fig 3 shows the percentage of each cluster in all of the instances. Six clusters are obtained from the features of inter-ICN PS. The micro-state is represented by the mean inter-ICN PS within the corresponding cluster. From Fig 2 and Fig 3, most of the dynamical PS between ICNs exhibits as state 2. Fig 2 and Fig 3 also show that states 3 and 4 are close to each other, while states 5 and 6 are close to each other. Fig 4 shows that, during state 2, most of ICNs behave in a dys-synchronized way. Fig 5 show that MVN, DMN and RFPN are more active than other ICNs, while CBN is the most quiet network.

### Altered temporal patterns of ICNs under EO/EC

**Fig 6:**
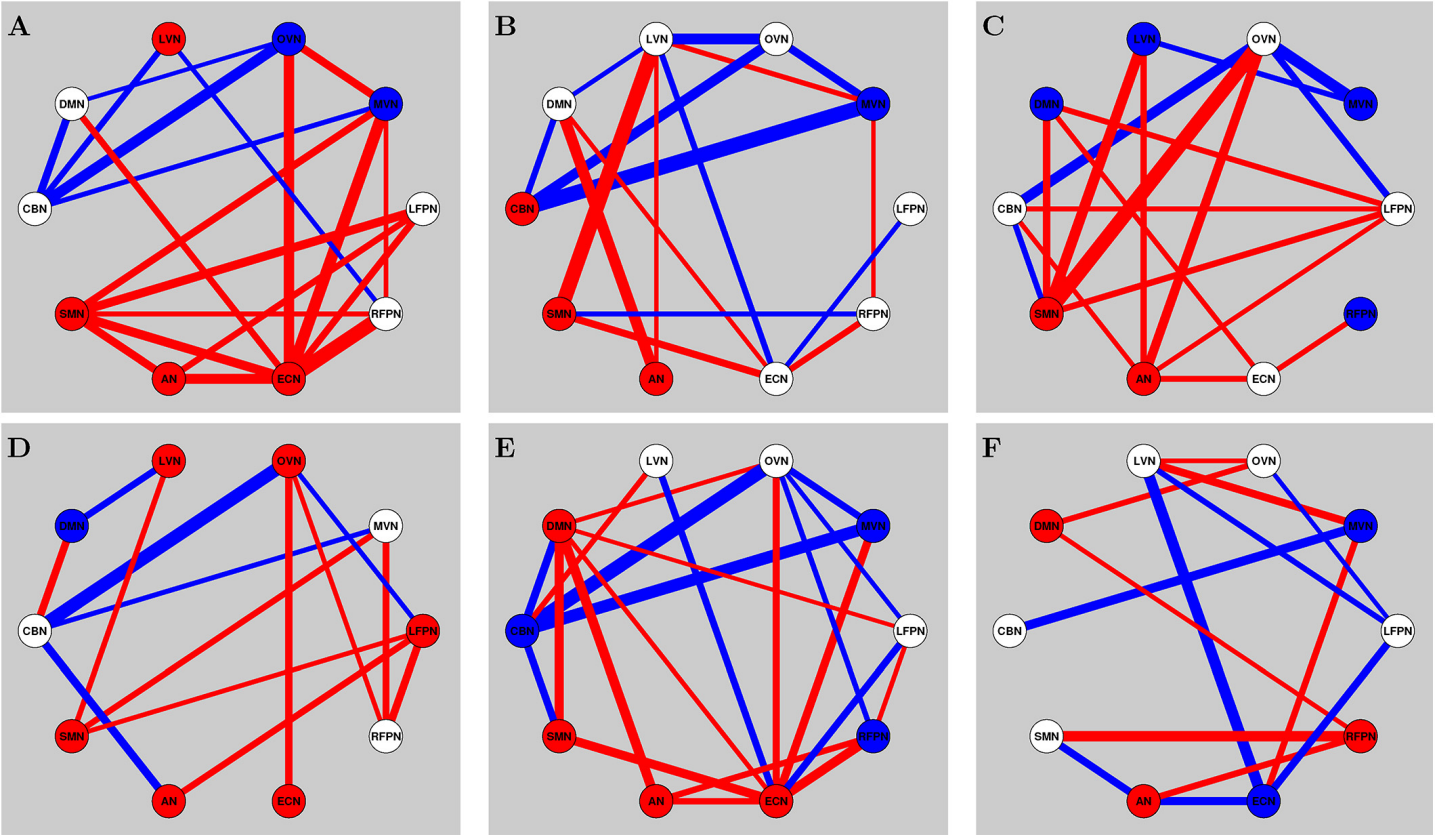
Altered inter-ICN dynamical PS and intra-ICN amplitude under EO and EC. Subfigures A, B, C, D, E and F denote states 1, 2, 3, 4, 5, and 6, respectively. Red lines denote increased PS during EC relative to EO. Blue lines denote decreased PS during EC relative to EO. Red circles denote increased amplitude during EC relative to EO. Blue circles denote decreased amplitude during EC relative to EO. (*p* < 0.001,FDR corrected).

Fig 6 shows the altered univariate and bivariate temporal patterns of ICNs under EO/EC (*p* < 0.001, FDR corrected). Here, red circles and lines denote the dynamical temporal patterns of ICNs are more active during EC relative to EO, while blue circles and lines indicate less active temporal patterns of ICNs during EC relative to EO.

From Fig 6, the MVN amplitude is decreased during EC for most of the micro-states, except for state 4. The amplitudes for SMN and AN are more active than other ICNs during EC than EO for most of the micro-states. The DMN exhibits altered amplitude during different resting states of EO/EC.

In particular, the results demonstrate altered dynamical inter-ICN PS during EO/EC. For most of the micro-states during EC, the cerebellum exhibits decreased PS to other ICNs. In addition, the RFPN, LFPN, and ECN mainly exhibited increased PS to other ICNs during EC. For micro-states 1 and 3 during EC, the SMN and AN exhibits enhanced PS to other ICNs. For micro-states 5 and 6 during EC, the DMN exhibits enhanced PS to other ICNs. For micro-states 1, 4 and 5 during EC, enhanced PS is found between OVN and ECN.

### Relationships of DMN amplitude and DMN strength under EO/EC

**Fig 7:**
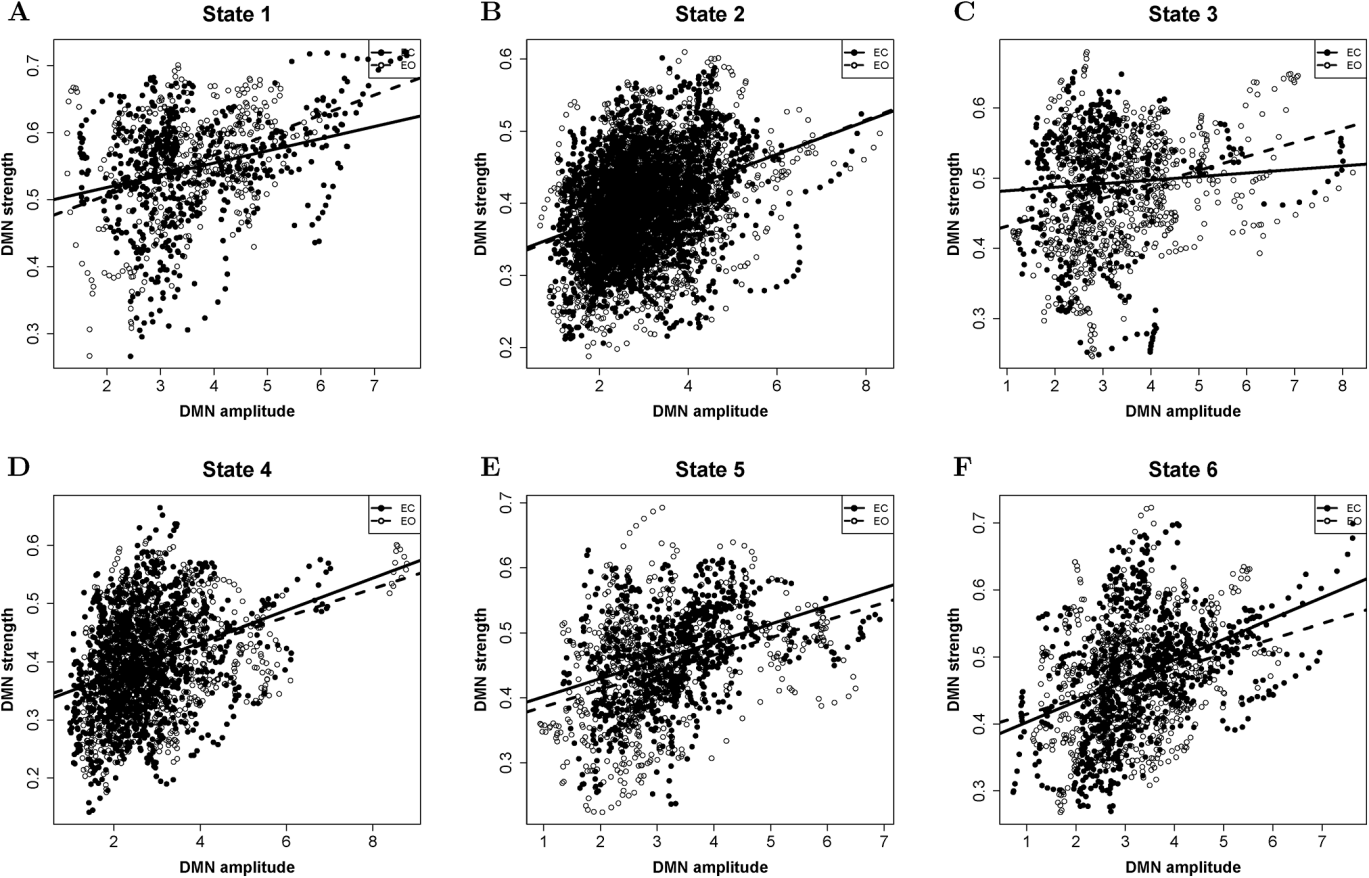
DMN amplitude correlates DMN strength of PS for EO and EC. Subfigures A, B, C, D, E and F denote states 1, 2, 3, 4, 5, and 6, respectively. Solid dots and lines represent EC-related resting state. Soft dots and dashed lines represent EO-related resting state.

Fig 7 presents the scatter plots of node amplitude and node strength for DMN. Table 2 shows the correlation coefficients and z-values under EO/EC. Certain linear relationships are existed between DMN amplitude and DMN strength. Different scan conditions of EO/EC might alter the positive correlation between DMN amplitude and DMN strength. For micro-state 2, there are no difference relationships of network amplitude and node strength during EO and EC. For micro-states 3, the correlations of DMN amplitude and DMN strength are decreased under resting state of EC. For micro-states 6, the correlations of DMN amplitude and DMN strength are increased under resting state of EC.

**Table 2:** Relationships of DMN amplitude and DMN strength.

State	EC (r, p)	EO (r, p)	EC-EO (z, p)
**State1**	(0.28, 0^a^)	(0.37, 0 ^a^)	(-1.56, 0.11)
**State2**	(0.33, 0 ^a^)	(0.36, 0 ^a^)	(-1.03, 0.3)
**State3**	(0.07, 0.15)	(0.3, 0 ^a^)	(-4.07, 0^b^)
**State4**	(0.31, 0 ^a^)	(0.33, 0 ^a^)	(-0.49, 0.63)
**State5**	(0.37, 0 ^a^)	(0.35, 0 ^a^)	(0.48, 0.63)
**State6**	(0.4, 0 ^a^)	(0.28, 0 ^a^)	(2.85,0^c^)

^a^ p<10^−10^

^b^ p<0.0001

^c^ p<0.01

### Altered relationships of DMN amplitude and inter-ICN PS under EO/EC

**Fig 8:**
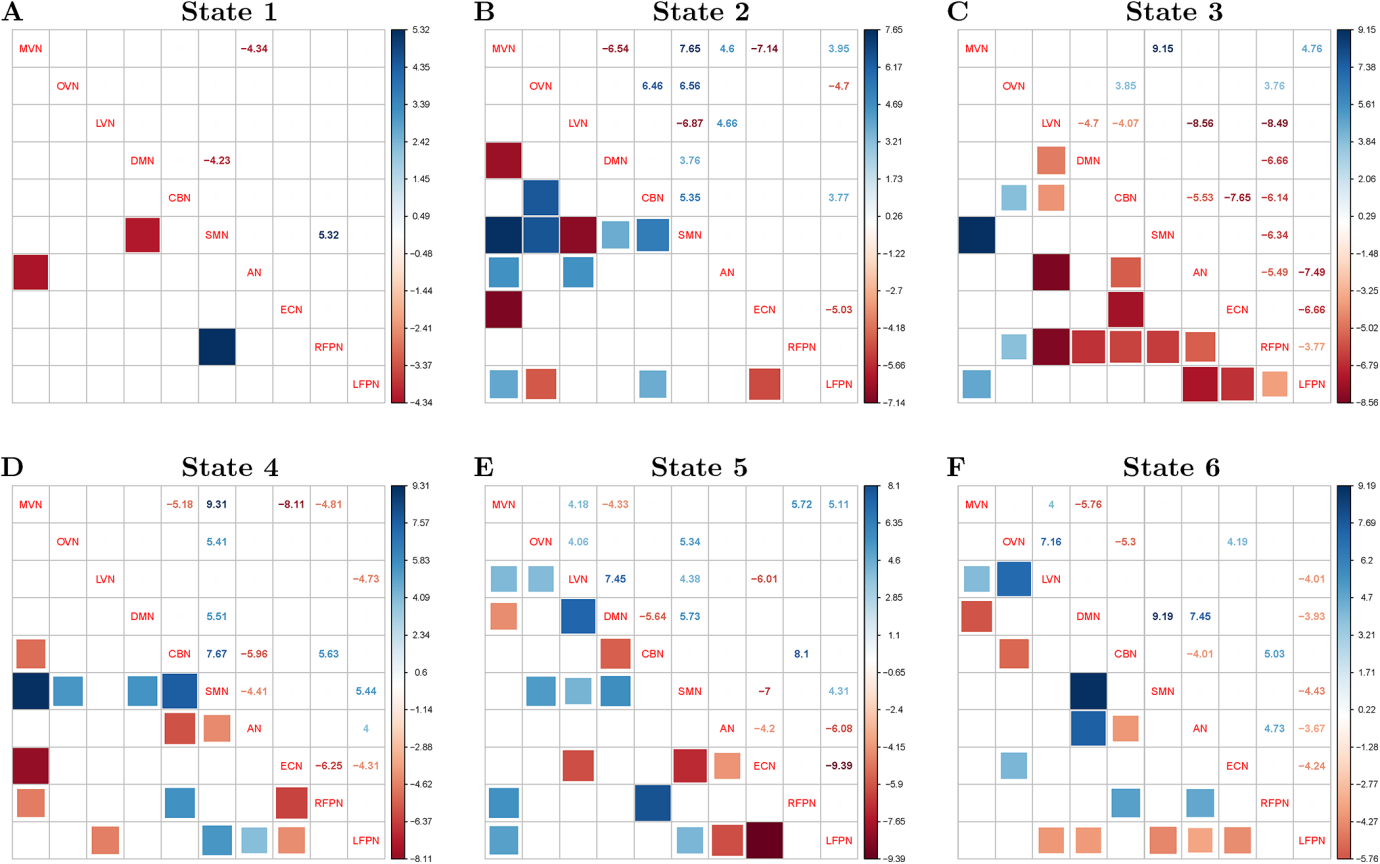
Altered relationships of DMN amplitude and inter-ICN PS under EO and EC. Subfigures A, B, C, D, E and F denote states 1, 2, 3, 4, 5, and 6, respectively. Blue rectangles represent increased linear relationships between DMN amplitude and network PS during EC relative to EO. Red rectangles represent decreased linear relationships between DMN amplitude and network PS during EC relative to EO. (*p* < 0.001,FDR corrected).

Fig 8 shows the altered relationships of DMN amplitude and inter-ICN PS during different scan conditions of EO/EC (*p* < 0.001, FDR corrected). The red squares denote increased linear correlations of DMN amplitude and inter-ICN PS during EO relative to EC, while blue squares denote decreased linear correlations of DMN amplitude and inter-ICN PS during EO relative to EC. For micro-state 1 during EO, the correlations of DMN amplitude and dynamic PS are increased between DMN and SMN, as well as between MVN and AN. For micro-states 2, 4 and 5 during EO, the correlations of DMN amplitude and dynamic PS are decreased between SMN and other ICNs. For micro-state 3 during EO, the relationships of DMN amplitude and dynamic PS are increased between RFPN and other ICNs. For micro-states 4 and 5 during EO, the correlations of DMN amplitude and dynamic PS are increased between ECN and other ICNs. For micro-state 6 during EO, the correlations of DMN amplitude and inter-ICN PS are increased between LFPN and other ICNs.

## Discussion

This paper aimed to investigate the different dynamical temporal patterns of ICNs under resting states of eyes-open/closed. To the best of our knowledge, this was the first attempt to simultaneously evaluate the dynamical univariate and bivariate features of neural signals in a unified framework of phase synchronization (PS). The results found altered intra-ICN amplitude and inter-ICN phase synchrony under EO/EC. In addition, the altered dynamical network amplitude might modulate network strength of synchronization. Furthermore, the dynamical network amplitude might correlate with inter-ICN phase synchrony. Together, dynamical univariate and bivariate features of neural signals could be investigated in the unified framework of Hilbert transform, and could be supplementary features to investigate the neurophysiological processes in human brain.

The dynamical states of functional coupling in human brain have been reported by resting state fMRI-based research. Different from conventional time-varying functional connectivity based on regional time-series, this study attempted to discover the dynamical states of ICN-related connectivity. Here, six micro-states of synchronization between ICNs were found via hierarchical clustering analysis. In resting state of EO/EC, over 40 percent of the ICN-related connectivities exhibited dys-synchronized patterns as state 2. This result confirmed previous findings that most of the ICNs were anti-correlated to each other based on stationary signal analysis [47]. Certain ICNs behaved in highly synchronized ways (i.e., state 1, state 3, state 5 and state 6) measured by time-varying synchrony. Conventional sliding-window technique also found there were unanticipated patterns of dynamical functional connectivity, compared to stationary functional connectivity [13]. In addition, state 3 and state 4 exhibited certain proximity, since they shared similar patterns of MVN and OVN-related inter-ICN PS according to supplementary analysis. The proximity of state 5 and state 6 might be related to the close patterns of inter-ICN PS among LVN, DMN and AN. The reason for the lower CBN amplitude might be its segregated function with less/slow connections to cortical regions [48]. In summary, the six states could be defined as follows: (1) state 1 might reflect highly synchronized state between ICNs; (2) state 2 could represent the highly dys-synchronized state between ICNs; (3) state 3 exhibited as visual and attention-related state; (4) state 4 might be vision-related state; (5) state 5 might be visual and auditory active state; and (6) state 6 might be auditory and attention-related state. Furthermore, we analyzed whether the results were robust against different sliding-windows. The additional findings exhibited certain tendencies of robustness against different length of sliding-window. States 1, 2, 4 and 6 appeared robustly with longer sliding-window. However, states 3 and 5 exhibited less robustness at certain length of sliding-window as outliers (i.e., state 3 at 34 TRs, state 5 at 28 TRs). One reason could be the criterions of similarity for pairs of states between different sizes of sliding-windows. Another reason might be the different numbers of instances between different sizes of sliding-windows. Nevertheless, the results of time-varying synchrony suggested the potential advantages of dynamical functional connectivity for research of brain mechanisms.

Eyes open or closed might be responsible for the exteroceptive or interoceptive resting state, respectively. In particular, EO was related to attention and ocular motor activity for exteroceptive state, while EC was related to imagination and multisensory activity for interoceptive state [2]. Additional analysis found higher global efficiencies of the dynamical states (i.e., states 1, 3 and 5) during EO compared to EC, implying the reorganized network during exteroceptive state [9, 49]. The high efficient organization of brain network during EO might be related to extra communications with external environment compared with EC [9]. However, lower global efficiency of inter-regional brain network under EO was found by resting state fMRI [49]. The inconsistent results of global efficiency between EO/EC might be related to different frequency bands (i.e., 0.01–0.08 Hz for fMRI, 4–45 Hz for MEG) or different types of graphs (i.e., inter-regional network, inter-ICN network). Previous study also argued that interoceptively or exteroceptivly oriented attention might exist in both EO and EC [49]. Furthermore, the global metrics of brain network topology were relatively invariant under different conditions of EO/EC [7]. Accordingly, no significantly different global efficiency was found in states 2, 4 and 6, suggesting that there might be ambiguous state besides exteroceptive or interoceptive state.

The dynamical intra-ICN amplitudes exhibited different patterns during resting state of EO/EC. The differences of brain active patterns under eyes open/closed have been reported by many resting state fMRI-related studies, most of which were voxel-wise measures (i.e., ALFF, regional homogeneity) [1]. Similar to decreased intra-ICN amplitude within SMN during EO, voxel-wise ALFF and regional homogeneity were also decreased within the sensory-motor regions during EO relative to EC [1, 50, 51]. Moreover, discriminative patterns of decreased fALFF under EO were found in sensorimotor regions through machine learning, suggesting sensorimotor regions may be vital for mental imagery [6]. Decreased high-frequency fluctuation were found in primary auditory and sensorimotor regions under EO, suggesting the cross-sensory modal inhibition process for exteroceptive state [4, 49]. In addition, our results found altered intra-ICN amplitude within visual-related ICNs under EO. Although there were increased local activities in visual cortex under EO [1, 50, 52], decreased signal variance, fALFF, as well as Hurst exponent were found in the primary and secondary sensory cortex under EO [7]. One reason for the contradictory results might be related to different visual inputs during EO [6, 10], since there were different written-word forms as visual stimuli [18]. Another reason might be related to different measures (i.e., intra-ICN ALFF, regional fALFF, voxel-wise CBF, regional homogeneity). Overall, dynamical intra-ICN amplitude could be prospective biomarker, which could determine the different temporal patterns of ICNs under EO/EC.

The dynamical inter-ICN phase synchrony also exhibited different patterns during resting state of EO/EC. Altered topological organization under EO/EC was found by graph-related research [49]. Characteristic directional connections were found under EO/EC using Bayesian network learning and support vector machine [10]. Here, sensory-motor and auditory-related inter-ICN synchronization were increased during EC compared to EO. The auditory network exhibited higher functional connectivity than other networks during EC compared to EO, evidenced by previous study [53]. One possible explanation could be the highly integrated sensory modalities during EC, suggesting that EO might inhibit cross-sensory modalities to allocate more resources for processing external world [49]. The huge information processing requirements during EO could be reflected by increased connection distance in all brain regions [7]. More directional connections related to primary visual network were found under EO compared to EC, implying EO was the exteroceptive state [10]. In this paper, increased visual-related inter-ICN PS was found under EO. Accordingly, higher efficient sensory-related ICNs were found in EO compared to EC, suggesting the need of more efficiently recruited sensory-related ICNs for exteroceptive information processing [9]. In summary, EO exhibited increased visual-related synchrony and suppressed sensorimotor and auditory-related synchrony for exteroceptive state. In addition, although increased synchronicity was found between visual system and attention system under EO [49], certain decreased synchronizations were found between visual networks and ECN, DMN, and RFPN during EO compared to EC in this study. One potential interpretation could be different measures of functional connectivity (i.e., PS, linear correlation coefficients). Another possible reason could be the time scales (44s) of dynamical features. Overall, these findings might provide additional information to investigate the different aspects of internal and external information processing in human brain [49].

This study also attempted to analyze the relationships of dynamical intra-ICN patterns and dynamical inter-ICN patterns. In this paper, the time-varying intra-ICN amplitude might reflect the dynamical information flows within each ICN. Meanwhile, the time-varying ICN strength might reflect the mean synchrony of the corresponding ICN to other ICNs. Our results found that intra-ICN amplitude might modulate network strength as well as inter-ICN synchrony under EO/EC differently. One previous study found characteristic relationships between intra-ICN complexity/ALFF and ICN strength based on stationary analysis [25]. Another study found local activity coincided almost exactly with resting state functional connectivity [52]. Specially, DMN could positively modulate the dynamical functional connectivity within the fronto-parietal network [15]. Furthermore, DMN was found as a hub network among the graph of ICNs [10]. In the inter-ICN graph, DMN exhibited relatively higher correlations of node amplitude and node strength than other ICNs. Compared to EO, decreased DMN amplitude was found in states 3 and 4, while increased DMN amplitude was found in states 5 and 6. Both increased and decreased node efficiencies corresponded to DMN were found in EO compared to EC across alpha and theta bands [9]. What is more, increased correlation of DMN amplitude and DMN strength was found in state 3 during EO relative to EC, and decreased correlation of DMN amplitude and DMN strength was found in state 6 during EO relative to EC. One possible interoperation could be states 3 and 6 were related to attention, in which DMN might play an important role [54]. Additionally, DMN could associate with the inter-ICN synchrony across different dynamical states in this study. In particular, the correlations between DMN amplitude and the SMN and visual-related inter-ICN synchrony were mainly decreased in the exteroceptive state than interoceptive state. According to recent evidence, the somato-sensory network, visual network as well as auditory network exhibited high node efficiencies as hubs during EO compared to EC [9]. Moreover, the enhanced correlations were found between DMN amplitude and the RFPN-related inter-ICN synchrony in state 3 during EO relative to EC. Increased correlations were found between DMN amplitude and the LFPN-related inter-ICN synchrony in state 6 during EO relative to EC. According to previous report, the RFPN and LFPN were related to perception and language respectively [18]. The above results implied that the perception and cognitive function could be modulated by changes of EO/EC [7]. Overall, the dynamics of DMN were related to multisensory system, perception, language, as well as other brain functions under EO/EC differently. Thus the dynamics of DMN might be fundamental for switching of exteroceptive state and interoceptive state.

One advantage of this study was simultaneously analyzing the dynamical intra-ICN and inter-ICN temporal patterns based on Hilbert transform. Thus, this study provided a unified mathematical framework to evaluate the dynamical functional connectivity within and between ICNs. Moreover, the intra-ICN amplitude and inter-ICN synchrony could reflect, to some extent, the differences of brain active patterns under resting state of EO/EC, implying the biological meanings of temporal patterns of ICNs. Another advantage of this study was the nonlinearity of PS, which outperforms the classical Pearson correlation coefficient-related measures. Moreover, the PS was an non-negative measure, ranging from 0 to 1. Thus, PS could capture the full information of functional connectivity between ICNs. Therefore, the proposed dynamical temporal patterns could be beneficial to ICN-related research, and could be potential biomarkers for brain disorder and brain development.

One limitation of this study was that we only considered resting states of eyes open/closed. As we known, other visual conditions (i.e., fixation) as well as external visual stimulus were also involved in resting states [7, 53]. Another limitation of this study was relatively small sample size of resting scans. Future studies should increase the sample size based on different populations. The third limitation was the fixed frequency band (i.e., 0.01–0.08Hz), which could be extended with sub-bands in future study [34, 55]. The results should also be verified by different scanners and scan parameters [4]. In particular, the impacts of EO/EC on the test-retest reliability should be considered by multi-sites dataset [5, 53, 56]. Furthermore, the metric of PS could not reflect the directional information between ICNs. Therefore, directional measures should be considered in future research [10]. Overall, the investigation of brain mechanisms among different visual conditions was an interesting topic, suggesting the need for further research.

## Conclusion

We demonstrated a unified framework to investigate the dynamical temporal patterns within and between ICNs under EO/EC. The ICNs exhibited different active patterns which were related to exteroceptive state and interoceptive state. Our results suggested that the scan conditions of eyes open/closed should be carefully considered when analyzing resting sate fMRI-related experiments. Furthermore, the proposed intra-ICN amplitude and inter-ICN phase synchronization could benefit the research of dynamical functional connectivity, and could also be prospective neural metrics for human functional connectome.

## Supporting Information

S1 Fig Mean dynamical inter-ICN PS with threshold of 0.5 for each cluster. In each subfigure, the names of ICNs are listed in the diagonal line. Subfigures A, B, C, D, E and F denote states 1, 2, 3, 4, 5, and 6, respectively. The inter-ICN PS below 0.5 is set to 0.
(TIFF)

S2 Fig Histogram for number of clusters. X-axis denotes number of clusters. Y-axis denotes the counts of number. The histogram is based on the median value of 1000 simulations of clustering.
(TIFF)

S3 Fig The impacts of sliding−window size on clusters. X-axis denotes the length of TRs. Y-axis denotes the correlation coefficients between the corresponding size of sliding−window and 44s sliding−window. The red, yellow, green, light blue, blue and pink curves denote states 1, 2, 3, 4, 5, and 6, respectively.
(TIFF)

S4 Fig The impacts of sliding−window size on temporal patterns of ICNs between EO/EC (state 1). Subfigures denote sliding-window length of 13 TRs,16 TRs, 19 TRs, 22 TRs, 25 TRs, 28 TRs, 31 TRs, 34 TRs, 37 TRs and 40 TRs respectively. Red lines denote increased PS during EC relative to EO. Blue lines denote decreased PS during EC relative to EO. Red circles denote increased amplitude during EC relative to EO. Blue circles denote decreased amplitude during EC relative to EO. (*p* < 0.001,FDR corrected).
(TIFF)

S5 Fig The impacts of sliding−window size on temporal patterns of ICNs between EO/EC (state 2). Subfigures denote sliding-window length of 13 TRs,16 TRs, 19 TRs, 22 TRs, 25 TRs, 28 TRs, 31 TRs, 34 TRs, 37 TRs and 40 TRs respectively. Red lines denote increased PS during EC relative to EO. Blue lines denote decreased PS during EC relative to EO. Red circles denote increased amplitude during EC relative to EO. Blue circles denote decreased amplitude during EC relative to EO. (*p* < 0.001,FDR corrected).
(TIFF)

S6 Fig The impacts of sliding−window size on temporal patterns of ICNs between EO/EC (state 3). Subfigures denote sliding-window length of 13 TRs,16 TRs, 19 TRs, 22 TRs, 25 TRs, 28 TRs, 31 TRs, 34 TRs, 37 TRs and 40 TRs respectively. Red lines denote increased PS during EC relative to EO. Blue lines denote decreased PS during EC relative to EO. Red circles denote increased amplitude during EC relative to EO. Blue circles denote decreased amplitude during EC relative to EO. (*p* < 0.001,FDR corrected).
(TIFF)

S7 Fig The impacts of sliding−window size on temporal patterns of ICNs between EO/EC (state 4). Subfigures denote sliding-window length of 13 TRs,16 TRs, 19 TRs, 22 TRs, 25 TRs, 28 TRs, 31 TRs, 34 TRs, 37 TRs and 40 TRs respectively. Red lines denote increased PS during EC relative to EO. Blue lines denote decreased PS during EC relative to EO. Red circles denote increased amplitude during EC relative to EO. Blue circles denote decreased amplitude during EC relative to EO. (*p* < 0.001,FDR corrected).
(TIFF)

S8 Fig The impacts of sliding−window size on temporal patterns of ICNs between EO/EC (state 5). Subfigures denote sliding-window length of 13 TRs,16 TRs, 19 TRs, 22 TRs, 25 TRs, 28 TRs, 31 TRs, 34 TRs, 37 TRs and 40 TRs respectively. Red lines denote increased PS during EC relative to EO. Blue lines denote decreased PS during EC relative to EO. Red circles denote increased amplitude during EC relative to EO. Blue circles denote decreased amplitude during EC relative to EO. (*p* < 0.001,FDR corrected).
(TIFF)

S9 Fig The impacts of sliding−window size on temporal patterns of ICNs between EO/EC (state 6). Subfigures denote sliding-window length of 13 TRs,16 TRs, 19 TRs, 22 TRs, 25 TRs, 28 TRs, 31 TRs, 34 TRs, 37 TRs and 40 TRs respectively. Red lines denote increased PS during EC relative to EO. Blue lines denote decreased PS during EC relative to EO. Red circles denote increased amplitude during EC relative to EO. Blue circles denote decreased amplitude during EC relative to EO. (*p* < 0.001,FDR corrected).
(TIFF)

S1 File Relationships of ICN amplitude and ICN strength. (DOC)

S1 Table Difference of global efficiency between EO and EC. (DOC)
