# Supplementary figures and images for: Investigating the Temporal Patterns within and between Intrinsic Connectivity Networks under Eyes-Open and Eyes-Closed Resting States: A Dynamical Functional Connectivity Study Based on Phase Synchronization
Source: PLoS One. 2015 Oct 15;10(10):e0140300. doi: 10.1371/journal.pone.0140300 (PMC4607488; doi:10.1371/journal.pone.0140300)

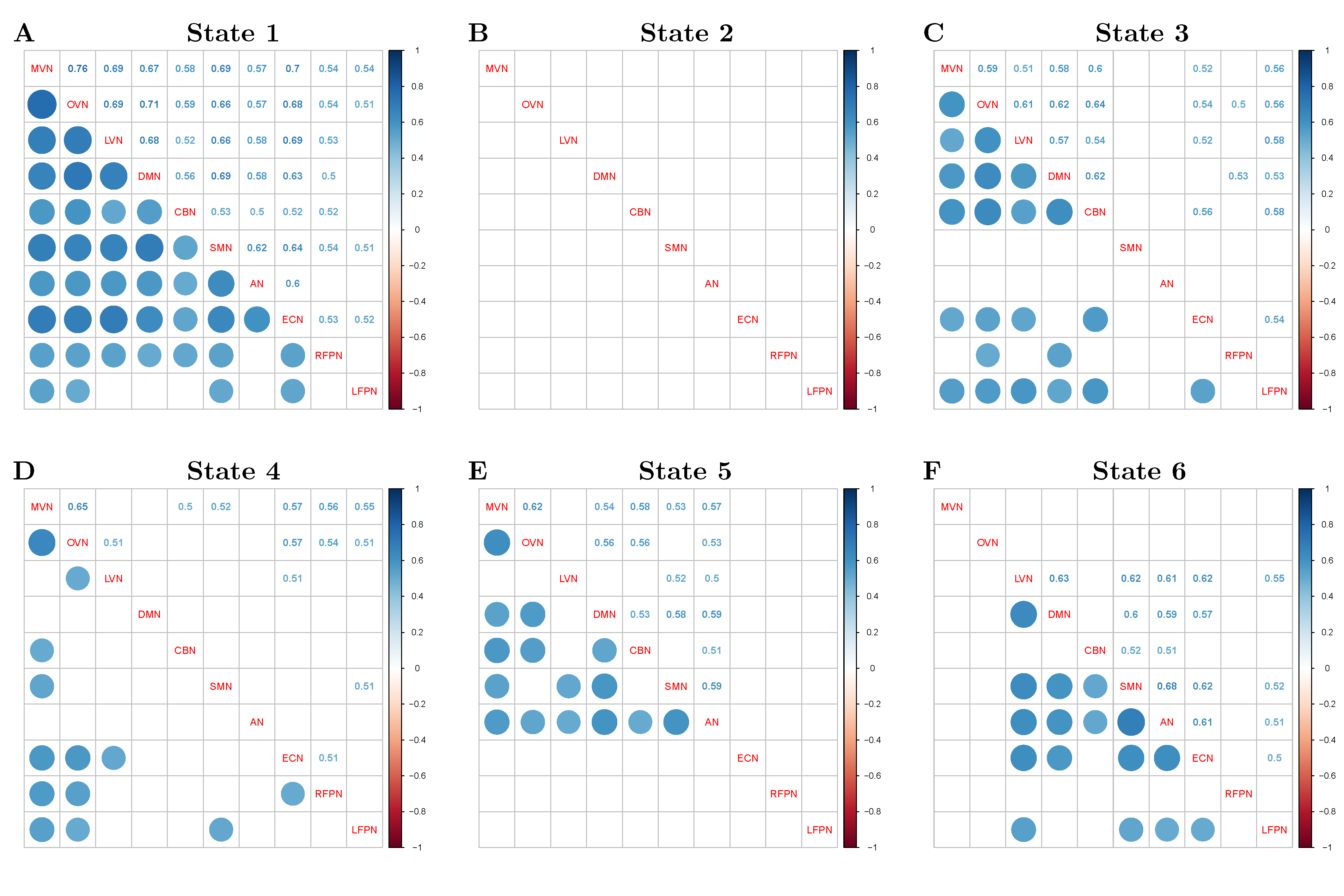

Supplement: S1 Fig — In each subfigure, the names of ICNs are listed in the diagonal line. Subfigures A, B, C, D, E and F denote states 1, 2, 3, 4, 5, and 6, respectively. The inter-ICN PS below 0.5 is set to 0. (TIFF) [file pone.0140300.s001.tiff]

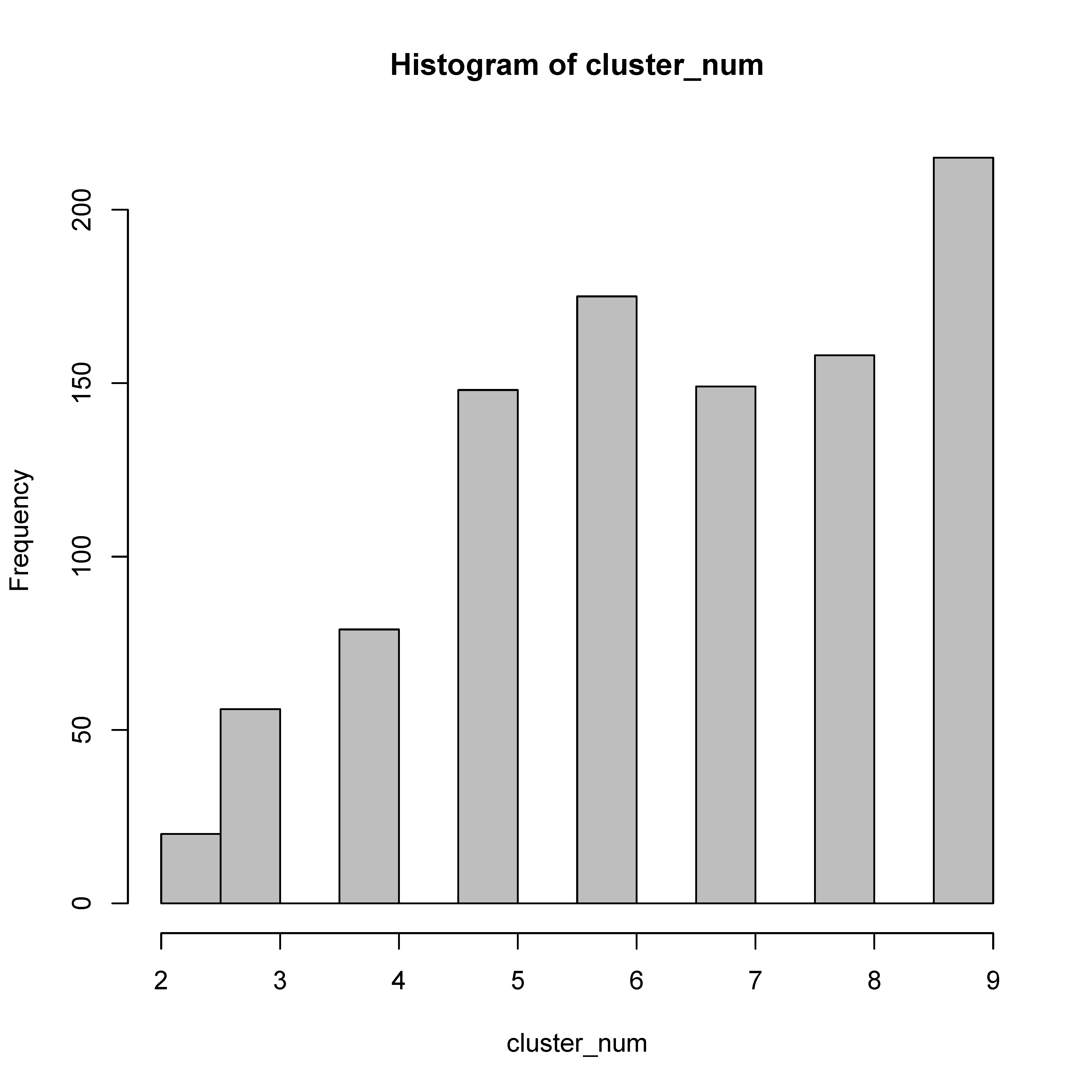

Supplement: S2 Fig — X-axis denotes number of clusters. Y-axis denotes the counts of number. The histogram is based on the median value of 1000 simulations of clustering. (TIFF) [file pone.0140300.s002.tiff]

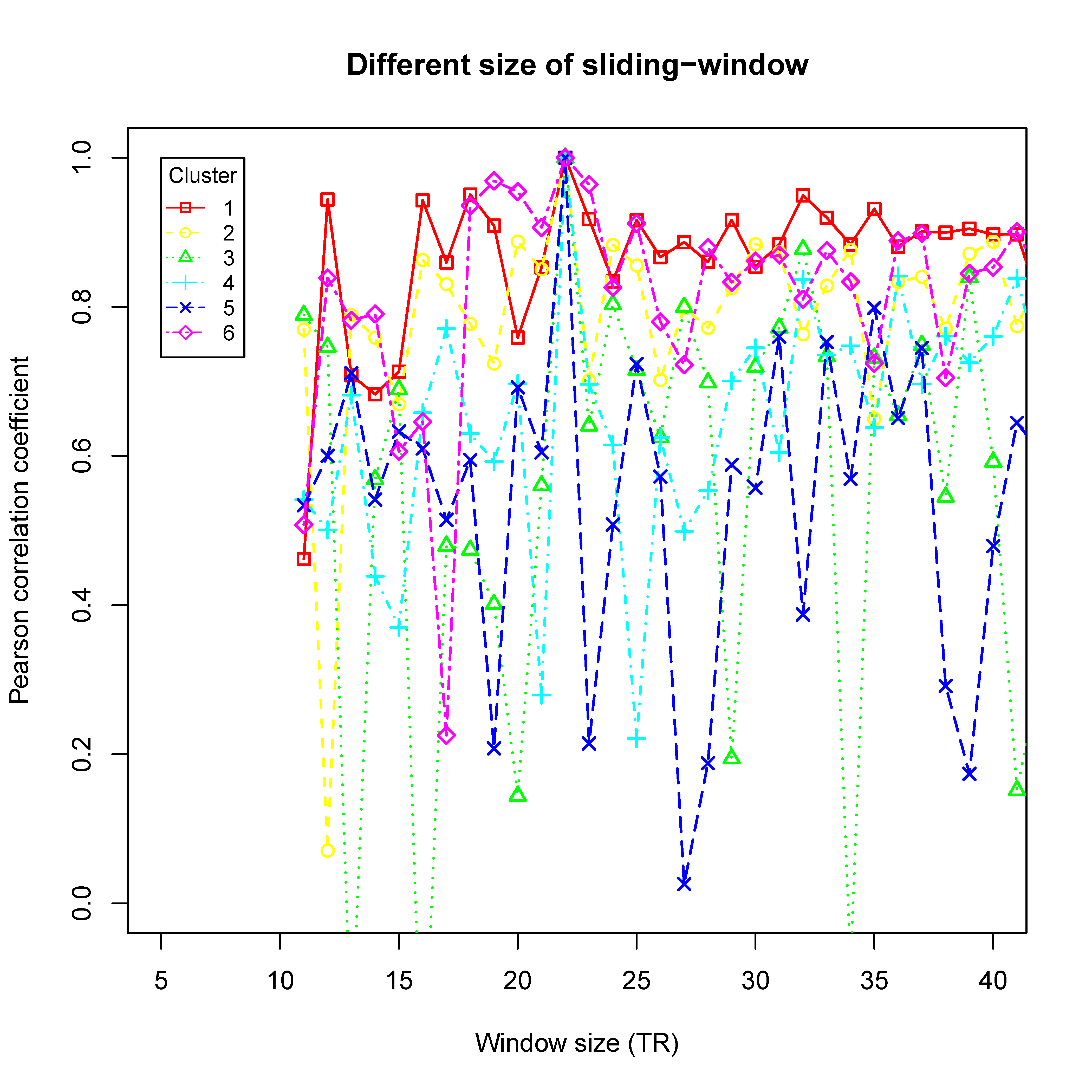

Supplement: S3 Fig — X-axis denotes the length of TRs. Y-axis denotes the correlation coefficients between the corresponding size of sliding−window and 44s sliding−window. The red, yellow, green, light blue, blue and pink curves denote states 1, 2, 3, 4, 5, and 6, respectively. (TIFF) [file pone.0140300.s003.tiff]

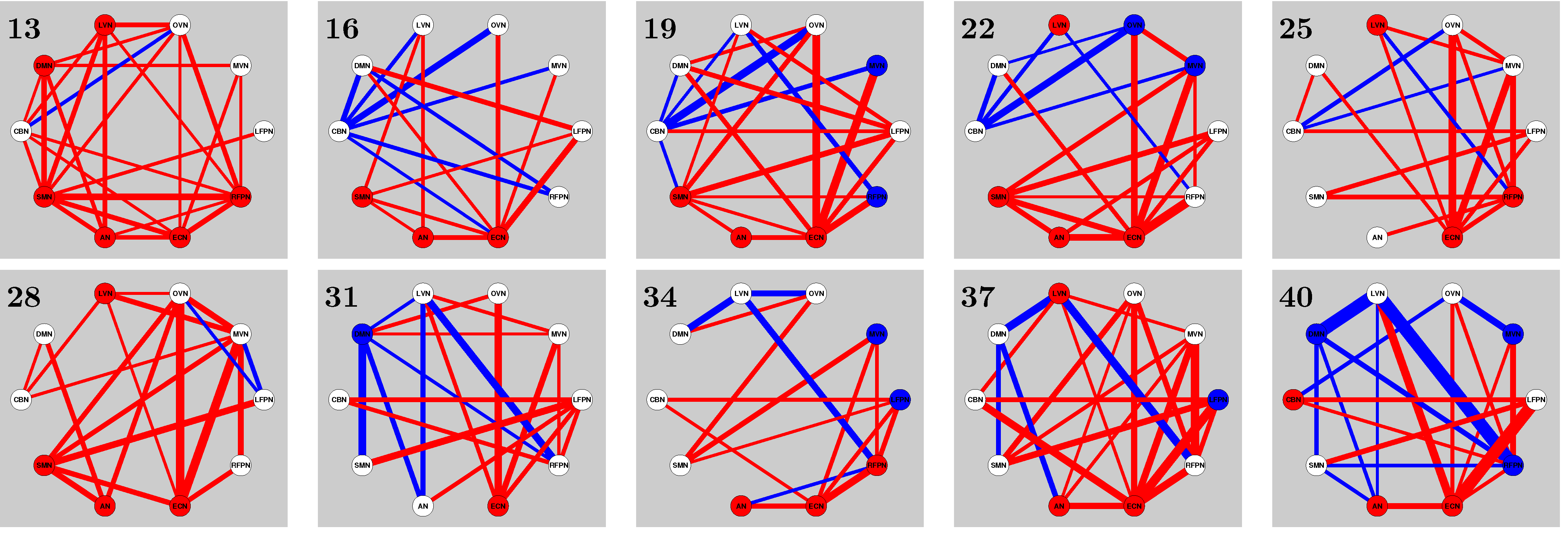

Supplement: S4 Fig — Subfigures denote sliding-window length of 13 TRs,16 TRs, 19 TRs, 22 TRs, 25 TRs, 28 TRs, 31 TRs, 34 TRs, 37 TRs and 40 TRs respectively. Red lines denote increased PS during EC relative to EO. Blue lines denote decreased PS during EC relative to EO. Red circles denote increased amplitude during EC relative to EO. Blue circles denote decreased amplitude during EC relative to EO. (p < 0.001,FDR corrected). (TIFF) [file pone.0140300.s004.tiff]

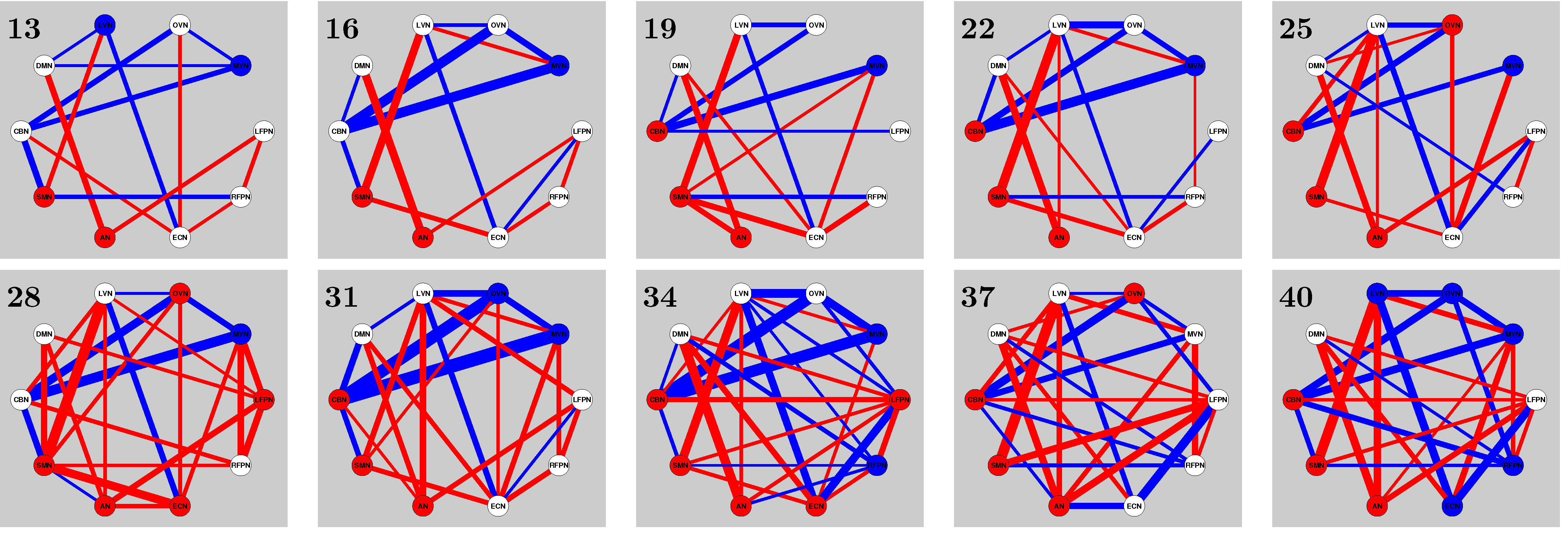

Supplement: S5 Fig — Subfigures denote sliding-window length of 13 TRs,16 TRs, 19 TRs, 22 TRs, 25 TRs, 28 TRs, 31 TRs, 34 TRs, 37 TRs and 40 TRs respectively. Red lines denote increased PS during EC relative to EO. Blue lines denote decreased PS during EC relative to EO. Red circles denote increased amplitude during EC relative to EO. Blue circles denote decreased amplitude during EC relative to EO. (p < 0.001,FDR corrected). (TIFF) [file pone.0140300.s005.tiff]

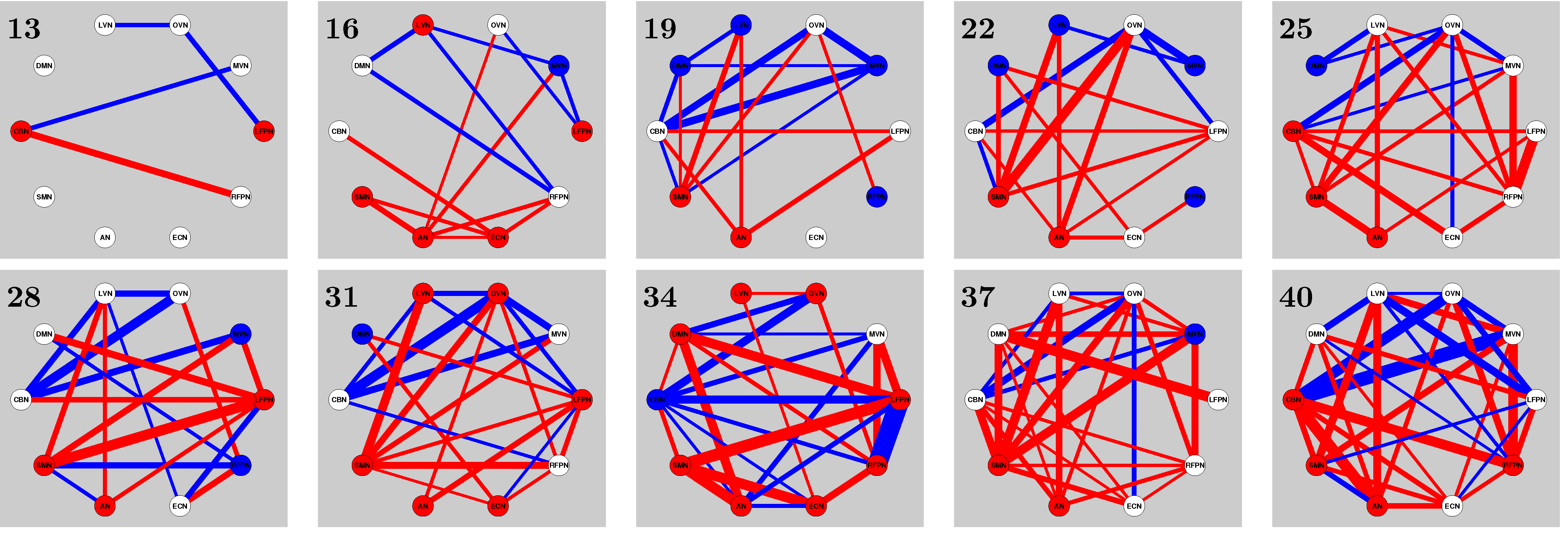

Supplement: S6 Fig — Subfigures denote sliding-window length of 13 TRs,16 TRs, 19 TRs, 22 TRs, 25 TRs, 28 TRs, 31 TRs, 34 TRs, 37 TRs and 40 TRs respectively. Red lines denote increased PS during EC relative to EO. Blue lines denote decreased PS during EC relative to EO. Red circles denote increased amplitude during EC relative to EO. Blue circles denote decreased amplitude during EC relative to EO. (p < 0.001,FDR corrected). (TIFF) [file pone.0140300.s006.tiff]

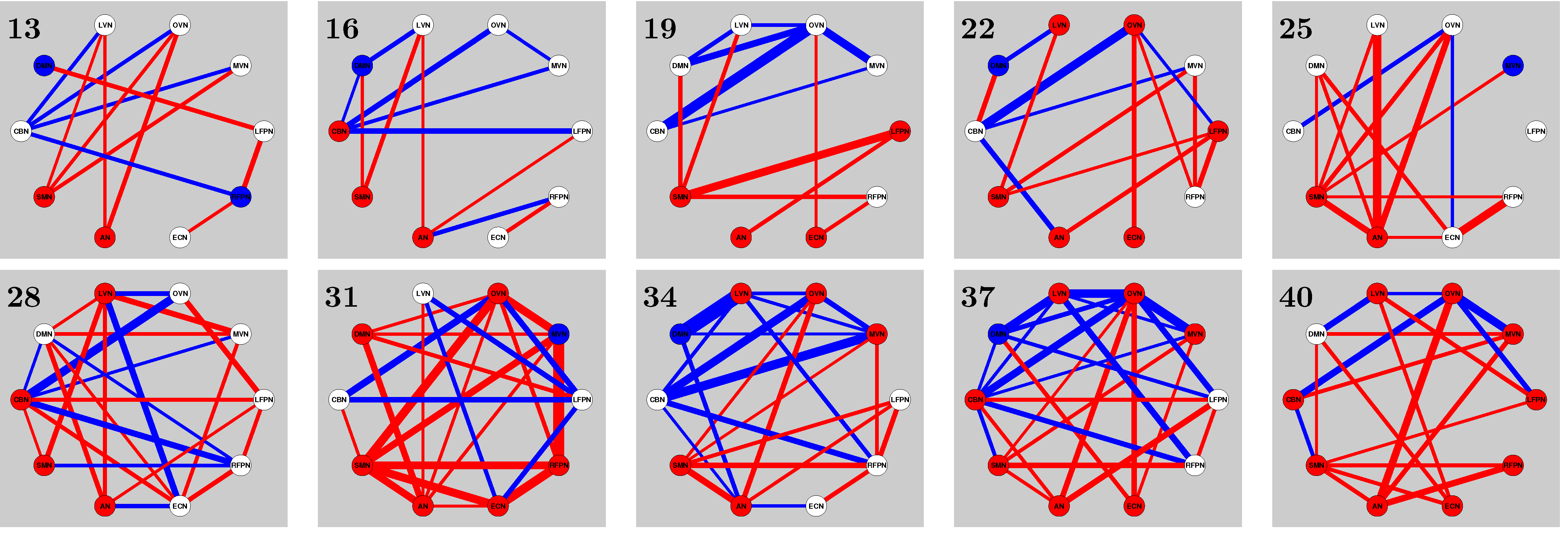

Supplement: S7 Fig — Subfigures denote sliding-window length of 13 TRs,16 TRs, 19 TRs, 22 TRs, 25 TRs, 28 TRs, 31 TRs, 34 TRs, 37 TRs and 40 TRs respectively. Red lines denote increased PS during EC relative to EO. Blue lines denote decreased PS during EC relative to EO. Red circles denote increased amplitude during EC relative to EO. Blue circles denote decreased amplitude during EC relative to EO. (p < 0.001,FDR corrected). (TIFF) [file pone.0140300.s007.tiff]

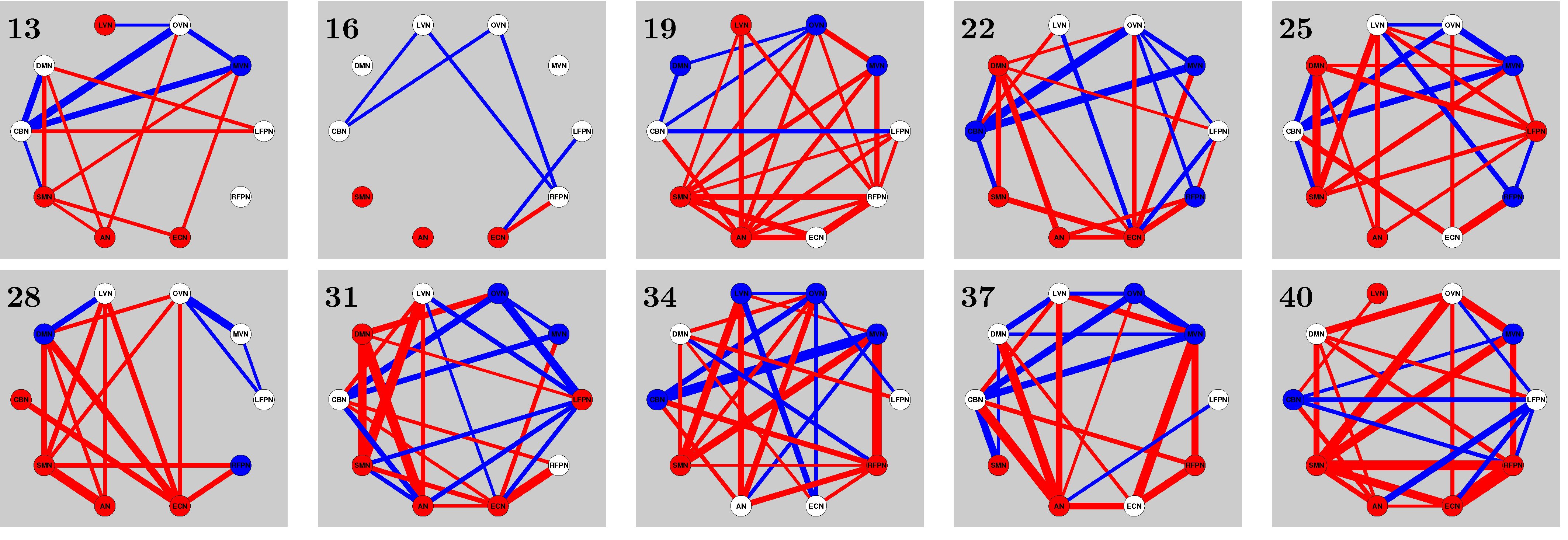

Supplement: S8 Fig — Subfigures denote sliding-window length of 13 TRs,16 TRs, 19 TRs, 22 TRs, 25 TRs, 28 TRs, 31 TRs, 34 TRs, 37 TRs and 40 TRs respectively. Red lines denote increased PS during EC relative to EO. Blue lines denote decreased PS during EC relative to EO. Red circles denote increased amplitude during EC relative to EO. Blue circles denote decreased amplitude during EC relative to EO. (p < 0.001,FDR corrected). (TIFF) [file pone.0140300.s008.tiff]

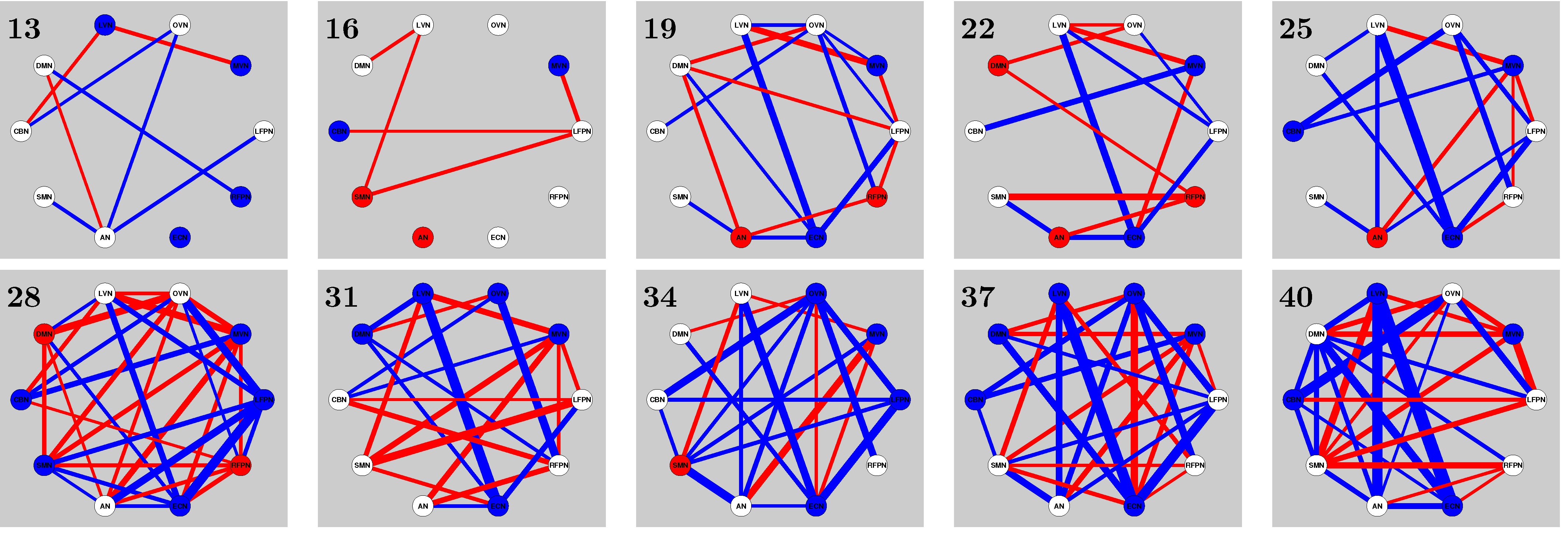

Supplement: S9 Fig — Subfigures denote sliding-window length of 13 TRs,16 TRs, 19 TRs, 22 TRs, 25 TRs, 28 TRs, 31 TRs, 34 TRs, 37 TRs and 40 TRs respectively. Red lines denote increased PS during EC relative to EO. Blue lines denote decreased PS during EC relative to EO. Red circles denote increased amplitude during EC relative to EO. Blue circles denote decreased amplitude during EC relative to EO. (p < 0.001,FDR corrected). (TIFF) [file pone.0140300.s009.tiff]
