# Supplementary material for: Investigating the Temporal Patterns within and between Intrinsic Connectivity Networks under Eyes-Open and Eyes-Closed Resting States: A Dynamical Functional Connectivity Study Based on Phase Synchronization
Source: PLoS One. 2015 Oct 15;10(10):e0140300. doi: 10.1371/journal.pone.0140300 (PMC4607488; doi:10.1371/journal.pone.0140300)
Supplement: S1 File — (DOC) [file pone.0140300.s010.doc]

Table A. Relationships of MVN amplitude and MVN strength

| State | EC (r, p) | EO (r, p) | EC-EO (z, p) |
| --- | --- | --- | --- |
| State1 | (0.18,0^b^) | (0.25, 0^c^) | (-1.08,0.28) |
| State2 | (-0.07,0^a^) | (0.18,0^d^) | (-9.49,0^d^) |
| State3 | (-0.25, 0^d^) | (-0.12, 0^a^) | (-2.29,0.02) |
| State4 | (0.16, 0^d^) | (0.05,0.11) | (2.76,0,01) |
| State5 | (0,0.84) | (0.1,0.01) | (-1.89,0.06) |
| State6 | (-0.04,0.29) | (-0.05,0.16) | (0.3,0.76) |

^a^p<10^-3^

^b^p<10^-5^

^c^p<10^-6^

^d^p<10^-7^

Table B. Relationships of OVN amplitude and OVN strength

| State | EC (r, p) | EO (r, p) | EC-EO (z, p) |
| --- | --- | --- | --- |
| State1 | (0.03,0.49) | (0.15, 0^a^) | (-1.87,0.06) |
| State2 | (-0.06,0^a^) | (0.17,0^e^) | (-8.6,0^e^) |
| State3 | (-0.12,0.01) | (0.1, 0^a^) | (-3.82,0^b^) |
| State4 | (0.16,0^c^) | (0.19, 0^d^) | (-0.83,0.4) |
| State5 | (0.10,0^a^) | (0.27, 0^e^) | (-3.4,0^b^) |
| State6 | (0,0.92) | (-0.12, 0^a^) | (2.4,0.02) |

^a^p<10^-2^

^b^p<10^-3^

^c^p<10^-7^

^d^p<10^-9^

^e^p<10^-12^

Table C. Relationships of LVN amplitude and LVN strength

| State | EC (r, p) | EO (r, p) | EC-EO (z, p) |
| --- | --- | --- | --- |
| State1 | (-0.09,0.03) | (0.31,0^f^) | (-6.33,0^f^) |
| State2 | (0.04,0.03) | (0.09,0^c^) | (-1.92,0.05) |
| State3 | (-0.23,0^d^) | (0.11,0^a^) | (-5.95,0^e^) |
| State4 | (0.12,0^b^) | (0.12,0^f^) | (-1.68,0.09) |
| State5 | (0.1,0.01) | (-0.03,0.4) | (2.54,0.01) |
| State6 | (0.12,0^a^) | (0.26,0^g^) | (-3.04,0^a^) |

^a^p<10^-2^

^b^p<10^-4^

^c^p<10^-5^

^d^p<10^-6^

^e^p<10^-8^

^f^p<10^-9^

^g^p<10^-12^

Table D. Relationships of CBN amplitude and CBN strength

| State | EC (r, p) | EO (r, p) | EC-EO (z, p) |
| --- | --- | --- | --- |
| State1 | (-0.14,0^b^) | (0.07,0.16) | (-3.22,0^a^) |
| State2 | (-0.07,0^b^) | (0.02,0.31) | (-3.33,0^b^) |
| State3 | (-0.28,0^8^) | (-0.05,0.15) | (-3.99,0^c^) |
| State4 | (0.1,0^b^) | (-0.04,0.16) | (3.51,0^c^) |
| State5 | (0.04,0.23) | (0.08,0.02) | (-0.85,0.4) |
| State6 | (0.06,0.07) | (-0.13,0^b^) | (3.86,0^b^) |

^a^p<10^-2^

^b^p<10^-3^

^c^p<10^-4^

^d^p<10^-8^

Table E. Relationships of SMN amplitude and SMN strength

| State | EC (r, p) | EO (r, p) | EC-EO (z, p) |
| --- | --- | --- | --- |
| State1 | (0.1, 0.01) | (0.21,0^a^) | (-1.76,0.08) |
| State2 | (-0.11,0^c^) | (0.02,0.23) | (-4.92,0^b^) |
| State3 | (-0.36,0^f^) | (0.02,0.53) | (-6.78,0^d^) |
| State4 | (0.2,0^e^) | (-0.07,0.02) | (6.63,0^d^) |
| State5 | (0.11,0.01) | (0.1,0.01) | (0.28,0.78) |
| State6 | (-0.08,0.02) | (-0.05,0.17) | (-0.61,0.54) |

^a^p<10^-4^

^b^p<10^-6^

^c^p<10^-8^

^d^p<10^-10^

^e^p<10^-12^

^f^p<10^-14^

Table F. Relationships of AN amplitude and AN strength

| State | EC (r, p) | EO (r, p) | EC-EO (z, p) |
| --- | --- | --- | --- |
| State1 | (-0.06,0.15) | (0.25,0^d^) | (-4.76,0^c^) |
| State2 | (-0.07,0^b^) | (-0.04,0.02) | (-1,0.31) |
| State3 | (-0.13,0.01) | (0.05,0.19) | (-2.95,0^a^) |
| State4 | (0.16,0^f^) | (0.02,0.41) | (3.39,0^b^) |
| State5 | (0.12,0^b^) | (0.21,0^e^) | (-1.7,0.09) |
| State6 | (0.03,0.41) | (0.07,0.06) | (-0.8,0.42) |

^a^p<10^-2^

^b^p<10^-3^

^c^p<10^-5^

^d^p<10^-6^

^e^p<10^-7^

^f^p<10^-8^

Table G. Relationships of ECN amplitude and ECN strength

| State | EC (r, p) | EO (r, p) | EC-EO (z, p) |
| --- | --- | --- | --- |
| State1 | (-0.14,0^b^) | (0.17,0^b^) | (-4.87,0^c^) |
| State2 | (0.05,0.01) | (-0.04,0.03) | (3.41,0^b^) |
| State3 | (-0.22,0^c^) | (-0.01,0.81) | (-3.69,0^b^) |
| State4 | (0.16,0^e^) | (0.11,0^b^) | (1.15,0.25) |
| State5 | (-0.1,0^a^) | (0.18,0^c^) | (-5.47,0^e^) |
| State6 | (0.12,0^b^) | (-0.18,0^d^) | (5.98,0^f^) |

^a^p<10^-2^

^b^p<10^-3^

^c^p<10^-5^

^d^p<10^-6^

^e^p<10^-7^

^f^p<10^-8^

Table H. Relationships of RFPN amplitude and RFPN strength

| State | EC (r, p) | EO (r, p) | EC-EO (z, p) |
| --- | --- | --- | --- |
| State1 | (-0.14,0^b^) | (0.1,0.04) | (-3.77,0^b^) |
| State2 | (0.01,0.72) | (0.15,0^g^) | (-5.26,0^d^) |
| State3 | (-0.27,0^f^) | (0.2,0^e^) | (-8.12,0^g^) |
| State4 | (0.13,0^c^) | (0.12,0^b^) | (0.33,0.74) |
| State5 | (-0.06,0.09) | (0.17,0^c^) | (-4.62,0^c^) |
| State6 | (-0.1,0^a^) | (0.01,0.7) | (-2.36,0.02) |

^a^p<10^-2^

^b^p<10^-3^

^c^p<10^-5^

^d^p<10^-6^

^e^p<10^-7^

^f^p<10^-8^

^g^p<10^-13^

Table I. Relationships of LFPN amplitude and LFPN strength

| State | EC (r, p) | EO (r, p) | EC-EO (z, p) |
| --- | --- | --- | --- |
| State1 | (0.06,0.15) | (0.13,0.01) | (-1.14,0.25) |
| State2 | (0.1,0^c^) | (0.07,0^b^) | (0.99,0.32) |
| State3 | (-0.35,0^e^) | (-0.01,0.78) | (-6,0^d^) |
| State4 | (-0.05,0.07) | (0,0.95) | (-1.22,0.22) |
| State5 | (0.02,0.63) | (-0.05,0.19) | (1.27,0.2) |
| State6 | (0.01,0.84) | (-0.12,0^a^) | (2.46,0.01) |

^a^p<10^-2^

^b^p<10^-3^

^c^p<10^-6^

^d^p<10^-8^

^e^p<10^-13^
