# Supplementary material for: Investigating the Temporal Patterns within and between Intrinsic Connectivity Networks under Eyes-Open and Eyes-Closed Resting States: A Dynamical Functional Connectivity Study Based on Phase Synchronization
Source: PLoS One. 2015 Oct 15;10(10):e0140300. doi: 10.1371/journal.pone.0140300 (PMC4607488; doi:10.1371/journal.pone.0140300)
Supplement: S1 Table — (DOC) [file pone.0140300.s011.doc]

Table S1. Difference of global efficiency between EO and EC

| State | EC-EO (T, p) |
| --- | --- |
| State1 | (-2.46, 0.01) |
| State2 | (0.4, 0.69) |
| State3 | (-2.82,0^a^) |
| State4 | (0.07, 0.94) |
| State5 | (-4.61,0^b^) |
| State6 | (-0.9,0.37) |

^a^p<10^-2^

^b^p<10^-5^
